# Traditional and non-traditional behavioral tests demonstrate the attenuation of cognitive deficits by therapeutic hypothermia in a rat model of neonatal hypoxic–ischemic encephalopathy

**DOI:** 10.3389/fnbeh.2025.1695435

**Published:** 2025-12-17

**Authors:** Angela Saadat, Cortney Kaszowski, Haree Pallera, Frank Lattanzio, Richard A. Britten, Asna Sulaiman, Stephanie Newman, Alireza Hosseini, Tushar Shah

**Affiliations:** 1 Department of Pediatrics, Macon & Joan Brock Virginia Health Sciences at Old Dominion University, Norfolk, VA, United States; 2 Neonatal Brain Institute, Norfolk, VA, United States; 3 Children's Specialty Group, Norfolk, VA, United States; 4 Children’s Hospital of the King’s Daughters, Norfolk, VA, United States; 5 Department of Biomedical and Translational Sciences, Macon & Joan Brock Virginia Health Sciences at Old Dominion University, Norfolk, VA, United States; 6 Department of Radiation Oncology and Biophysics, Old Dominion University, Norfolk, VA, United States; 7 Center for Integrative Neuroscience and Inflammatory Disease, Old Dominion University, Norfolk, VA, United States; 8 School of Health Professions, Macon & Joan Brock Virginia Health Sciences at Old Dominion University, Norfolk, VA, United States

**Keywords:** cognitive behavioral tests, sex-specific effects, hypoxic–ischemic encephalopathy, therapeutic hypothermia, attention, hyperactivity

## Abstract

**Introduction:**

Neonatal hypoxic-ischemic encephalopathy (HIE) is neurological disease caused by the deprivation of oxygen and blood flow to the brain during the developmentally-critical perinatal period. Therapeutic hypothermia (TH) is the standard of care treatment for HIE, though cognitive deficits can persistent throughout life despite treatment. The nature of these deficits, and the impacts of TH and sex are not well understood, and this presents a key barrier in the development of novel therapeutics.

**Methods:**

The goal of this study was to enhance the characterization and measurement of cognitive outcomes with tasks that measure spontaneous behaviors in a rodent models of HIE. Mild-moderate HIE was induced in term-equivalent rats by Vannucci’s method and a subset of rats were treated with TH. Cognitive performance was assessed between 6-12 weeks of age.

**Results:**

Hyperactivity and topographical disorientation were observed in HIE rats. Injured rats also spent less time investigating a novel object, suggesting HIE reduced their ability to encode or recognize a familiar object and switch attention to a new object. In a food protection test, injured rats failed to detect an approaching robber rat and protect food items, an indication of impaired attention and egocentric spatial processing. TH treatment resulted in sex-specific attenuation of deficits in attention, learning and skill acquisition, feeding, and processing self-centered spatial cues.

**Discussion:**

These observations highlight the need for deeper understanding of the enduring social and cognitive consequences of neonatal HIE including cases where therapeutic hypothermia was administered. This can pave the way for the development of tailored interventions that enhance the ability of HIE survivors to navigate the complex social and cognitive landscape of adult life.

## 1 Introduction

Neonatal hypoxic–ischemic encephalopathy (HIE) is caused by the deprivation of oxygen and blood flow to the brain during the developmentally critical perinatal period. The subsequent brain injury is variable and depends on a number of factors (Greco et al., 2020; Thornton et al., 2017; Northington et al., 2011), though damage to subcortical and cortical regions is common (Kebaya et al., 2023; Li et al., 2022; Douglas-Escobar and Weiss, 2015). Functionally, HIE is associated with increased risks of cognitive and motor impairments. These cognitive outcomes include attention deficit, autism spectrum disorder, anxiety, depression, and difficulties with social interactions; these impairments can be debilitating and persist into adolescence and adulthood (Kromm et al., 2024; van Handel et al., 2010; Schreglmann et al., 2020). The standard of care treatment for HIE is therapeutic hypothermia (TH) (van Oldenmark et al., 2024), which offers an estimated 11% reduction in risk of death or disability (Robertson et al., 2012).

Progress toward adjuvant treatments for HIE is hindered in part by difficulties in measuring cognitive effects in animal models (She et al., 2023). We therefore set out to characterize a set of cognitive tests that we and others can use to measure long-term outcomes in our animal model of HIE. For this, we chose a test not previously used in HIE animal studies, food protection, as well as tests historically used in HIE, novel object recognition (NOR), and open field exploration. We chose this specific mixture of tests because they 1—rely on spontaneous behavior, 2—overlap in functional assessments, and 3—are economical and straightforward to conduct. Since many cognitive processes initiate spontaneously, such as attention fluctuation and memory activation (Kucyi et al., 2018), we reasoned that behavioral tests that rely on spontaneous, naturally occurring behaviors can allow more sensitive measurement of these cognitive disruptions. Further, the overlap between novel and established tests enables us to anchor novel insights in the food protection assay alongside those already established in the field. We emphasized movement in outcome measures in each test, since movement is a highly organized process (Ohgi et al., 2008) and deficits in processing the cues that guide movement in spontaneous behaviors can lead to topographical disorientation and difficulties in temporal processing (Wallace, 2017).

The goal of this study was to enhance the characterization and measurement of cognitive outcomes of neonatal HIE modelled in rats. To investigate this, we induced mild–moderate HIE in term-equivalent rat pups and treated a subset with TH. Following, their cognitive performance was assessed in an open field exploration (Osterlund Oltmanns et al., 2021; Kraeuter et al., 2019), novel object recognition (Antunes and Biala, 2012; Cohen et al., 2013; Mathiasen and DiCamillo, 2010) and food protection (Wallace et al., 2006; Peters, 2018). Injured rats demonstrated deficits in attention and processing self-movement cues. The TH treatment mitigated some deficits, some of which were sex-specific; it was found to introduce an atypical behavior in learning. A nuanced understanding of cognitive dysfunction in HIE can lead to improved diagnostic accuracy, enhanced prognostic capabilities, and targeted therapeutic interventions, as well as help explain the variability observed in HIE outcomes.

## 2 Methods

### 2.1 Animals

Timed-pregnant Wistar rats were procured from Hilltop Lab Animals, Inc. (Scottsdale, PA, USA) at embryonic day 19, housed individually, and allowed to deliver spontaneously. At postnatal day 1 (P1), the pups were pooled and randomly redistributed among the dams to control for litter effects. At P20, the pups were re-housed into same-sex pairs and weaned to Teklad rat chow. The rats were housed in the Comparative Medicine facility at Brock Virginia Health Sciences at Old Dominion University in a temperature and humidity-controlled room (68–76 °F, 30%–60%) with a 12-h diurnal light/dark cycle and treated in accordance with the Institutional Animal Care and Use Committees (IACUC) protocol #19–014. The rats were euthanized at ~13 weeks by Fatal Plus injection (150 mg/kg) and cardiac puncture.

### 2.2 Hypoxic–ischemic injury

**Figure 1:**
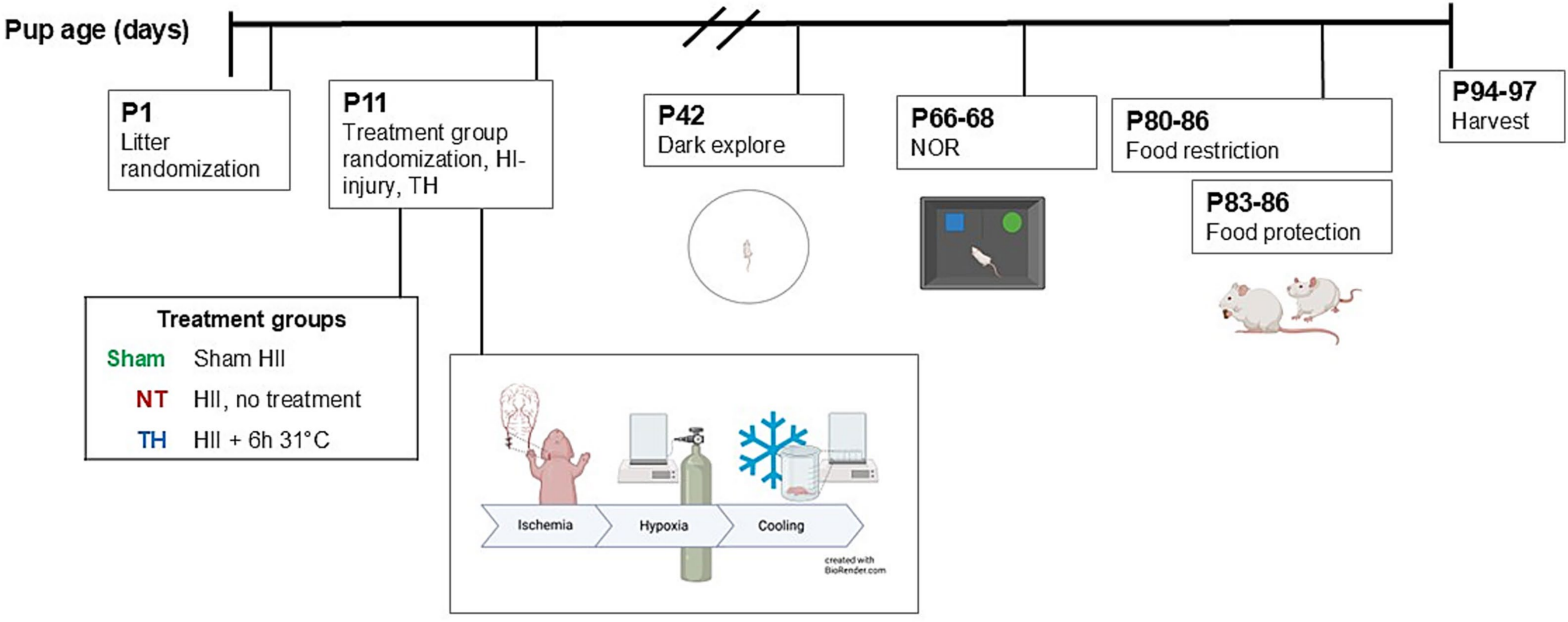
Experimental overview. Pups were randomized at P1 to control for litter effects, then again on the day of injury to treatment groups: Sham injury, NT (HI-injury and no treatment), and TH (HI-injury and therapeutic hypothermia). At P11, ischemia was induced by (permanent) ligation of the right carotid artery, followed by exposure to hypoxic conditions (8% O_2_ for 50 min with 10 min ramp from ambient). TH was 6 h 31 °C. Rats performed behavioral tests P42 to P86, first open field exploration; then NOR, then food protection, which required food restriction prior to and during testing. The rats were then harvested at P94-97. Created in BioRender (Saadat, 2025, q84v973 and q84v973, https://BioRender.com/).

At P11, the pups were randomized to one of three groups: Sham (no HI-injury control), NT [hypoxic–ischemic (HI)-injury and normothermia/no treatment], and TH (HI-injury and therapeutic hypothermia; Figure 1). Mild–moderate HIE was induced in the NT and TH rats using Vannucci’s method (Vannucci and Back, 2022) as we have described previously (Saadat et al., 2023; Shah et al., 2021). Briefly, pups were anesthetized by isoflurane inhalation (3% induction, 1% maintenance), and ischemia was induced by permanent ligation of the right common carotid artery (4–0 silk suture) at two sites approximately 5 mm apart. Sham rats received anesthesia, incision, and dissection but no ligation. Incisions were sealed with VetBond™, and each animal was allowed to recover (10–15 min) before returning to the dam for a 1-h rest period. To induce hypoxia, the ligated animals were placed in a custom chamber (BioSpherix) with 8% oxygen (balanced with nitrogen gas) and heated to 37 °C for 60 min (included in this time is a ~10 min period where the chamber returns to 8% O_2_ after being opened to place the animals inside). To control for this time away from the dam, Sham rats were placed in an identical chamber at ambient oxygenic conditions at 37 °C. One animal died following surgery.

### 2.3 Therapeutic hypothermia

Following a 1-h rest period post-hypoxia, pups in the TH group were placed in a chamber at 30 °C ± 2 °C for consecutive 6 h, where they maintained rectal temperatures 31 °C ± 1 °C (Shah et al., 2021; Shah et al., 2017). To control for this time away from the dam, Sham and NT rats were placed in a chamber at 37 °C, away from their dams.

### 2.4 Behavioral tests

#### 2.4.1 Open field exploration

The open field test is an established behavioral assay that exploits rodents’ natural inclination to explore a novel environment (Kraeuter et al., 2019). At 6 weeks of age, the rats underwent open field exploration test. For this, each rat was placed alone in the center of a round, plastic table (1.22 m diameter) and allowed 12 min to explore the field. Each rat was carried to the table in a circuitous path to impede its ability to establish a spatial anchor. The testing room was illuminated by two small infrared lights to encourage exploration (Thompson et al., 2018) and tests were filmed from a top–down view with a Canon XA30 camcorder. Behavior was tracked in the latter 10 min with ANY-maze software (Stoelting). Rats and other animals establish a home base in new environments, a specific, “safe” location they return to between exploration periods. Two min has been observed to be a sufficient duration of time for rodents to establish a home base (Eilam and Golani, 1989; Blankenship et al., 2017; Donaldson et al., 2018; Donaldson et al., 2019), and this time was excluded from analysis to not dilute the measurement of the target behaviors. The table was thoroughly cleaned with 70% ethanol and dried between rats to decrease odor cues.

#### 2.4.2 Novel object recognition

The NOR test is a simple, non-stressful, and relatively robust test for nonspatial memory in rodents that similarly exploits rodents’ innate exploration of novel objects in their environment (Mathiasen and DiCamillo, 2010; Cohen and Stackman, 2015). NOR was assessed when the rats were 9 weeks of age and spanned 3 consecutive days. On day 1, the rats were habituated to the testing chamber, a 61 cm × 61 cm × 46 cm black box, by exploring the empty chamber for 5 min. The following day, rats spent 5 min in the chamber with two identical objects (toy ducks). Each object was affixed to adjacent corners of the box and held in place with duct tape. On the third day, rats spent 5 min with the two familiar objects (FO), rested in their cage for 6 min, then spent 2 min with one FO and one novel object (NO, toy dinosaur). Room lighting was dimmed to approximately 4 Lux, and each trial was recorded from above with a video camera (Logitech HD) controlled by ANY-maze software (Stoelting). The apparatus and objects were cleaned with 70% ethanol between sessions and rats. Time spent with the object was defined as the amount of time the animal’s nose was detected within an approximately 2-cm perimeter around the object.

#### 2.4.3 Food protection

Food protection behavior in rodents is spontaneous and incorporates spatial self-awareness, the ability to calculate the time needed to consume the food item, and the coordination of motor initiation (Peters, 2018). In this test, a “victim” rat is given a food item and therefore tries to protect it from theft by the “robber” rat; the robber rat is not given the food item and therefore tries to steal it from the victim rat (Peters, 2018). Food protection behavior was tested when the rats were 11–12 weeks of age.

##### 2.4.3.1 Food restriction

Mild food restriction began 3 days prior to the start of food protection testing and ended the same day testing was completed. (The rats had unlimited access to food before and after this test.) The purpose of the restriction was to motivate the rats to protect and consume food items in the testing procedure. First, baseline body mass was established for each rat by averaging its mass over 3 consecutive days. After determining baseline body mass, rats were given a measured, daily allowance of rat chow. The allowance on the first day was 6 g per rat (i.e., 12 g per cage pair), and the allotted food weight thereafter was dictated by the percentage of weight lost over each 24-h period: 6 g was allotted if their weight was equal or less than 10% of their baseline weight or 10 g if between 11 and 15%. No rat lost more than 13% of their baseline body weight.

##### 2.4.3.2 Habituation

Rats were habituated to accept food items from stainless steel forceps daily for 3 days prior to the start of the testing trials.

##### 2.4.3.3 Testing procedure

Food protection testing was conducted over 4 consecutive days, with 3–5 trials for each rat per day. Test trials were filmed with a high shutter-speed camcorder (Canon XA30) aimed at a mirror placed on the floor beneath the testing apparatus, which was a clear, plastic 69 cm × 41 cm × 35 cm tub. For testing, two rats of the same sex, one “robber” and one “victim,” were placed together in the testing apparatus. Using the forceps, the victim rat was given a food item, and behavior was assessed as the victim tried to consume it and protect it from the robber rat. One trial equaled the consumption of one food item. Stolen items were returned to the victim rat to complete the trial if they were 30% or more of the starting size; if between 5% and 30% of the starting size, the rat was given half of a new item to finish the trial (<5% was considered complete). This continued until the food item was consumed, and the cycle was repeated 3–5 times each day for 4 days. All rats in the cohort were tested as the victim rat each day. The apparatus was cleaned with 70% ethanol as needed and between rat pairs. On day 4, after completing its testing as a victim rat, each rat was given a food item to consume alone (without the robber rat) for consumption time measurements. The rats were returned to free-feeding at the conclusion of the food protection trials.

In addition to the number of steals by the robber rat, the number of dodges and braces was also tallied. Dodges were defined as instances in which the food item was held in the victim rat’s mouth while it used its limbs to move away from the robber rat (Cohen et al., 2013). Braces were defined as instances where the food item was braced in the victim rat’s forepaws as it pivoted its body away from the robber rat (Cohen et al., 2013). Sham rats served as both robbers and victim rats, and were tested against each other at the start of each testing day. Only Sham animals served as robber rats. To differentiate them from the victim rat and from each other, Sham rats were uniquely marked with 1–2 lines on their abdomens.

##### 2.4.3.4 Food item

The food items used here were two Honey Nut Cheerios fused together (by wetting each and drying them together, on testing day 1) or half a Teddy Graham™ (testing days 2–4). For use in our model, the criteria we used for selecting the food items included that 1—the item be small enough that even the most injured rats were able to carry the item [HIE often induces motor weakness (Douglas-Escobar and Weiss, 2015), and was evident in this cohort (Saadat et al., 2023)], 2—yet large enough to allow sufficient time for food protection behavior demonstration, and 3—all rats would readily consume the item. Beans, nuts, and cereal items have all been used previously by other groups, ranging in mass from ~0.2 to 1 g (Peters, 2018). Pilot testing eliminated their Teklad chow (not readily appealing), nuts (dense and difficult to break into uniform pieces), and generic fruit loop and cookie cereals (not appealing to all the rats).

### 2.5 Histology

Following cardiac puncture, the rats were perfused with 100–200 mL phosphate-buffered saline (pH 7.38) and 50–100 mL 4% buffered formalin. Brains were harvested and imaged (Samsung AQ100 camera), then a ~2-mm slice was excised between −2.5 and −5 mm from the line of Bregma. The slices were paraffin-embedded, sectioned (5 μm), and hematoxylin and eosin (H&E)-stained at the EVMS Biorepository and Histology Lab in Norfolk, VA, USA. The leftover brain halves and H&E-stained sections were scanned (Epson Perfection V39). Tissue loss in the ischemic hemisphere and its structures were measured from images of the whole brain, coronal scans, and H&E-stained sections in ImageJ software (Schneider et al., 2012). For this, gates were drawn around each hemisphere, as well as the hippocampi and cortices. Percent reduction was calculated as 1 − (right area/left area), or 1 − (smallest area value/largest) for the stained sections, since the anatomical left and right were unknown following slide mount. To be clear, these injury assessments were previously published by Saadat et al. (2023), where results of behavioral tests assessing motor function were presented.

### 2.6 Statistical analyses

Throughout, the Sham group contained 7 animals (4 females and 3 males), the NT group contained 12 (6 females and 6 males), and the TH-treated group contained 10 animals (4 females and 6 males). All animals were tested, no rat values were excluded, and all datasets were analyzed with JMP version 17.2.0. To determine whether mean or median-based tests were appropriate, datasets were assessed for normality by Shapiro–Wilke’s and Anderson–Darling tests. Normal datasets were analyzed for equal variances [determined by *p* < 0.05 in one or more of four tests (O’Brien, Brown-Forsythe, Levene, Bartlett)]. The following datasets were assessed for group differences by one-way analysis of variance [shortened to analysis of variance (ANOVA) throughout] if the dataset followed normal distribution and equal variances, Welch’s ANOVA, if the dataset was normally distributed and had unequal variances, or by the Kruskal–Wallis (KW) method if the dataset was not normal. *Post hoc* Games–Howell (GH) and Dunnett’s (tested with both NT or Sham set as control) tests were used for parametric data sets, since these tests allow for unequal sample sizes, and by Steel–Dwass (SD) or Wilcox’s method (tested with both NT or Sham control) for non-parametric datasets. Significance was established as alpha < 0.05 throughout. Group and individual difference *p*-values, as well as the tests used, are listed in Supplementary Table 1.

## 3 Results

### 3.1 Open field exploration

**Figure 2:**
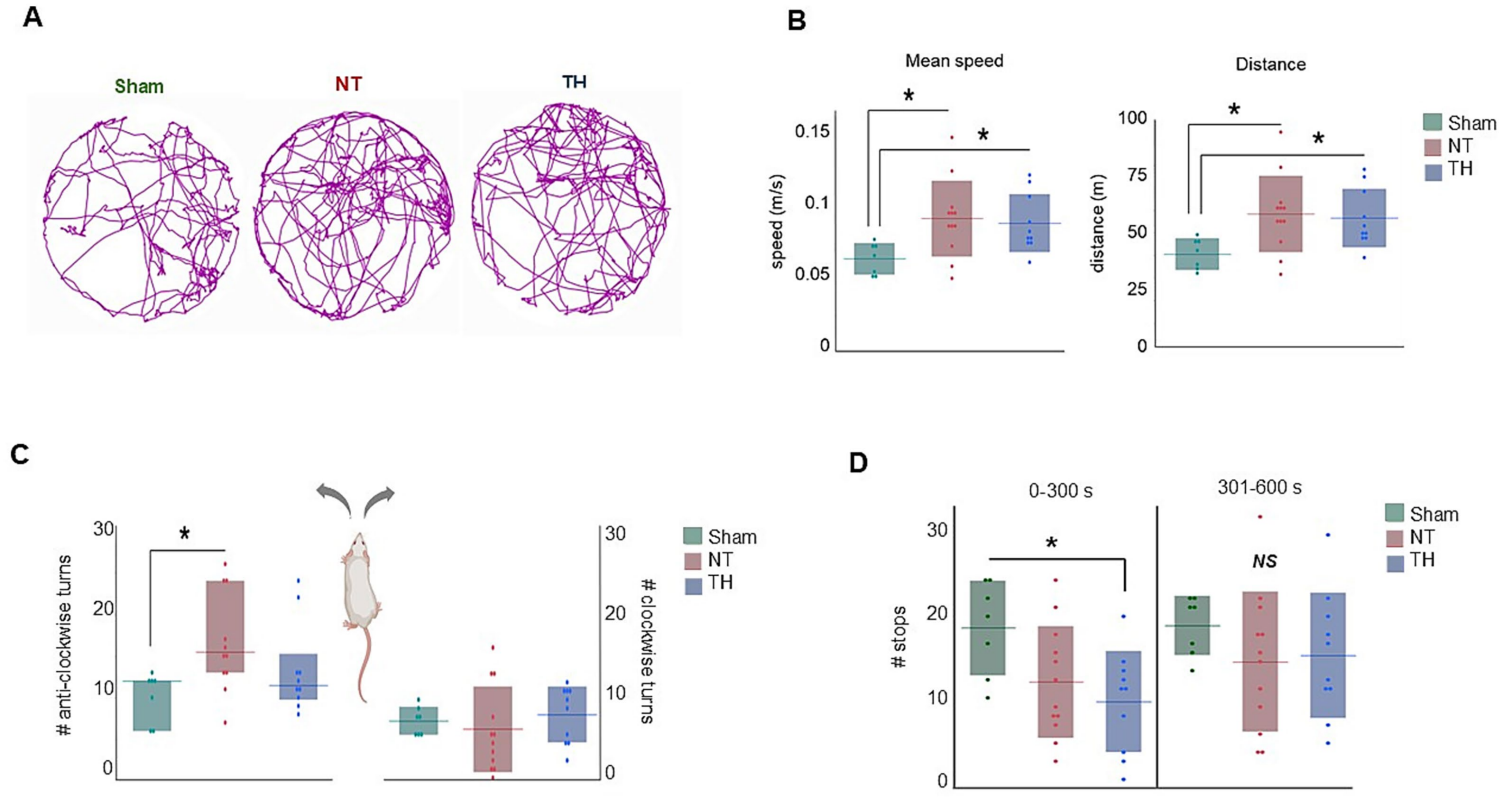
Open field exploration: **(A)** Representative track plots for each treatment group over the 10-min testing period. **(B)** Mean speed and distance were higher in injured animals compared to Sham rats. **(C)** NT rats made more anti-clockwise turns than Sham rats, whereas the number of anti-clockwise turns in TH rats was not different from either group. **(D)** The number of stops in the first 5-min half of exploration (0–300 s) was lower in TH-treated animals than Sham, while stops were not different among treatments in the second half of exploration (301–600 s). *p < 0.05 > 0.01. Floating horizontal bars represent means in **(B**,**D)**, clockwise turns in **(C)**, and medians in the anti-clockwise turns in **(C)**. Shaded bars represent standard deviation in **(B**,**D)**, and the clockwise turns in **(C)**, and interquartile range in the anti-clockwise turns graph in **(C)**. Dots represent individual rat values. Rat image in C created with BioRender (Saadat, 2025, q84v973, https://BioRender.com/).

**Figure 3:**
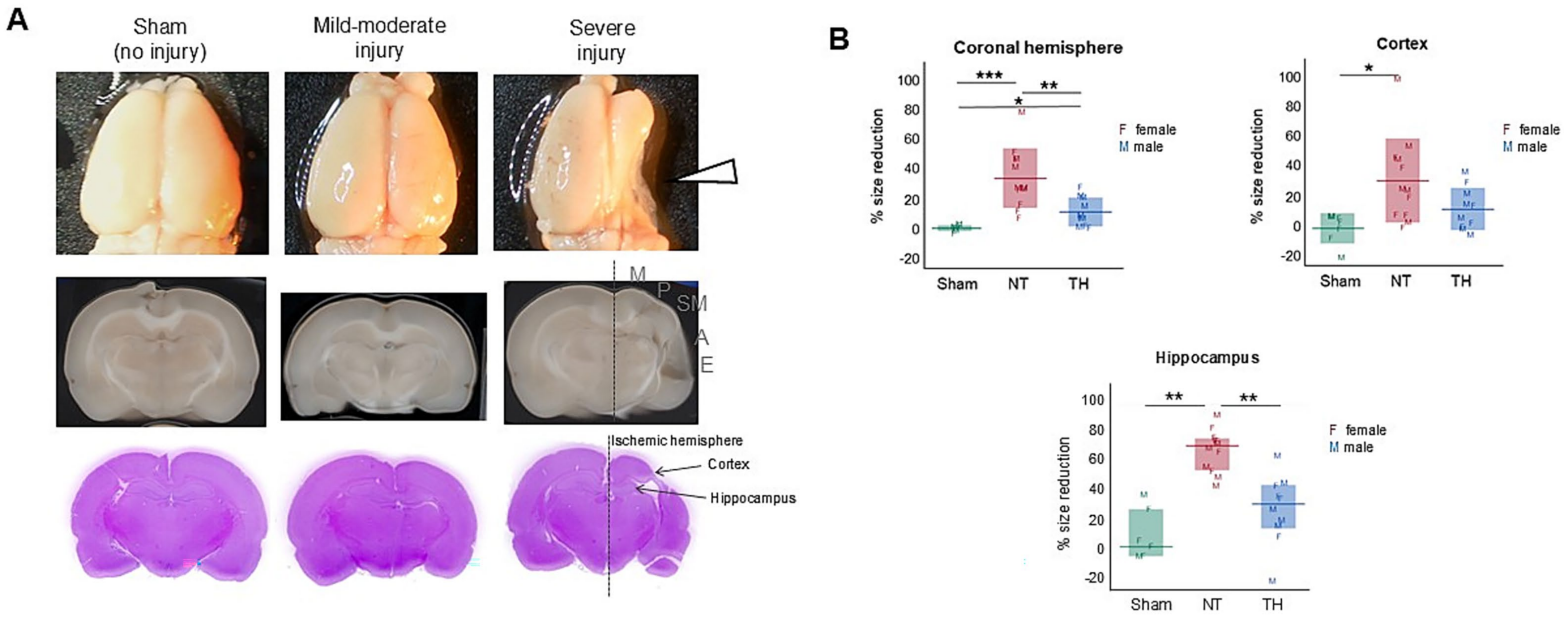
Cerebral injury from HIE was characterized by tissue loss in the ischemic hemisphere. **(A)** Representative images of whole brains (top), coronal scans (middle), and H&E-stained sections (bottom). The right hemisphere is ipsilateral to the carotid ligation and demonstrated variable injury, which was focused on ~−3 mm from the Bregma (indicated by the white arrowhead). Cortical injury was mainly to the sensorimotor area (indicated by the gray sensorimotor SM letters), but spanned variably from motor (M) to auditory regions (A), and rarely to the ectorhinal cortex (E). **(B)** Size reduction of the ischemic hemisphere, cortex, and hippocampus, relative to the contralateral hemisphere. Left and right hemispheres, cortices, and hippocampi were traced in ImageJ (Schneider et al., 2012) and size reduction in the ischemic hemisphere was calculated by dividing the ischemic hemisphere (right) area by the left hemisphere area and subtracting this product from 1. To be clear, the hemispheric, hippocampal, and cortical loss data were previously used in a model of injury published in Saadat et al. (2023) and are shown here to demonstrate the location and magnitude of structural injury. The measurements shown are from this exact cohort of rats. Hemisphere and cortex graphs show means (horizontal bars) and standard deviation (shaded bars), and the hippocampus graph shows medians and interquartile range. Individual rat values are shown as letters F and M, where F represents females and M represents males. *p < 0.05; **p < 0.005; ***p < 0.0005.

During the 10-min testing period, the injured rats (NT and TH) traveled a higher average speed and distance compared to Sham rats [(Figures 2A,B) speed: ANOVA *F*(2,26) = 4.0917, *p* = 0.02875; GH: *p* = 0.0140, 0.0141 Sham vs. NT and TH, respectively; distance: ANOVA *F*(2, 26) = 4.0824, *p* = 0.0287; GH: *p* = 0.0144, 0.0138 Sham vs. NT and TH, respectively]. Such hyperactive locomotion is an established consequence of hippocampal injury (Thompson et al., 2018), and injury to the hippocampus was observed in this cohort [previously published in Saadat et al. (2023) and shown here for demonstrative purposes in Figures 3A,B; KW H(2) = 19.7328, *p* < 5.2e-5; SD, *p* = 0.0011 NT vs. Sham]. NT rats made more anti-clockwise turns than Sham rats during the test, and the number of anti-clockwise turns in TH rats was not different from either group [Figure 2C; KW H(2) = 8.2241, *p* < 0.0164; SD, *p* = 0.0204 NT vs. Sham, *p* = 0.6792, 0.1917 TH vs. Sham and NT, respectively]. This higher proportion of turns to the left side of the body is likely a consequence of injury to the right cerebral hemisphere and contralateral weakness. This injury to the right hemisphere, which is ipsilateral to the ischemia induced by the carotid ligation in this experiment, was generally evident in injured rats collectively, but less so in TH-treated rats [previously published in Saadat et al. (2023) (Figures 3A,B) ANOVA *F*(2,26) Welch’s *p* = 5.9e-5, GH, *p* = 0.0003 NT vs. Sham, *p* = 0.0079 TH vs. NT]. Injured rats also engaged in less stops and progressions than Sham rats, although this was only significant in TH animals during the first half of exploration time [Figure 2D; stops: ANOVA *F*(2,25) = 3.4669, GH *p* = 0.0220; progressions: ANOVA F(2,26) = 4.0842, GH, *p* = 0.0265]. This significance in TH but not NT animals may be because the TH group contained two less females, and female rats were found to stop more often than males (14.7 mean stops vs. 13.0 over 10 min, respectively). Abnormalities in stops and progressions during exploration are associated with lesions to the hippocampus and structures connecting it to subcortical afferents, and reflect an inability to establish or recall the location of a home base (Travis et al., 2010).

### 3.2 Novel object recognition

**Figure 4:**
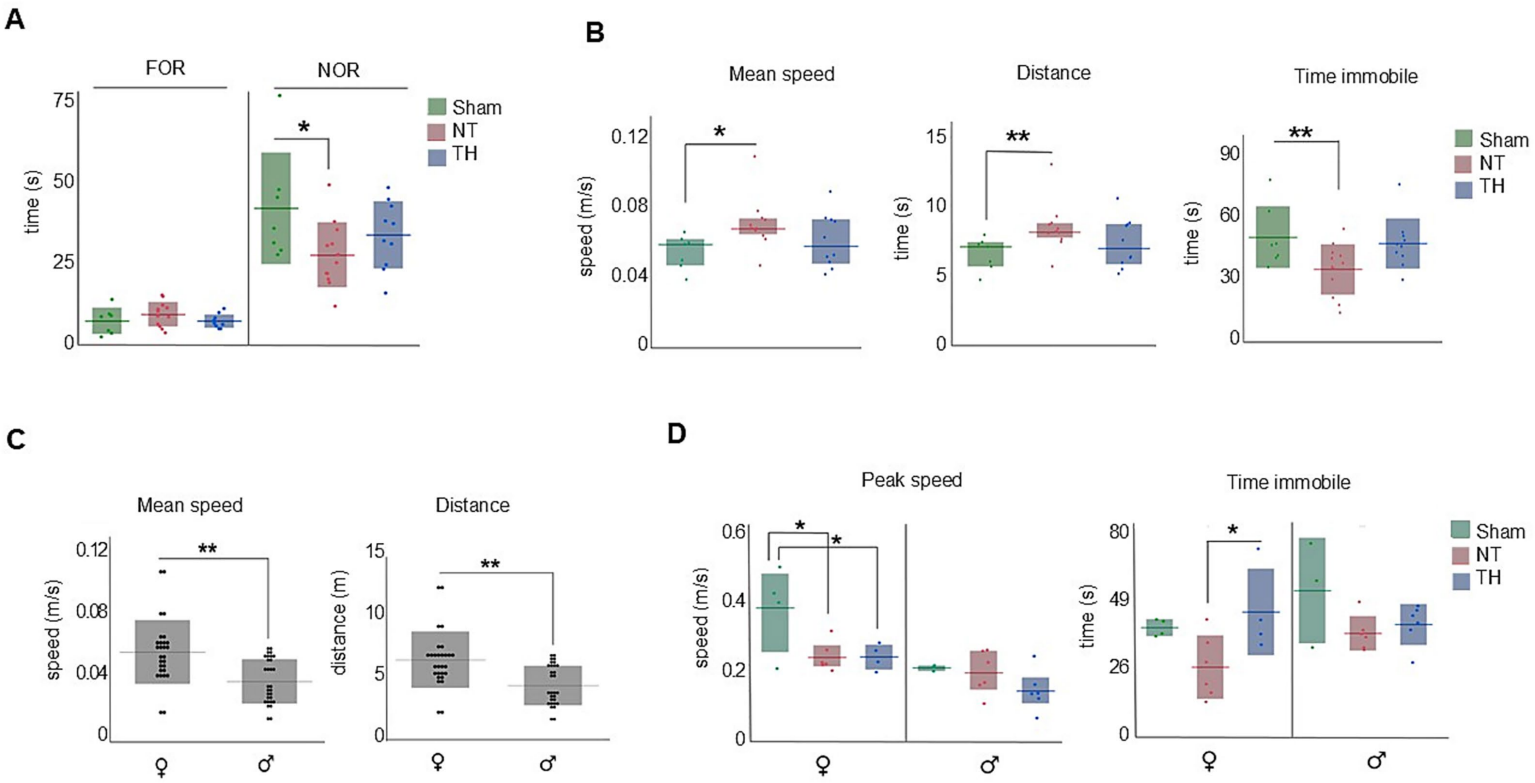
Novel object recognition (NOR). Non-spatial memory and measures of attention were impaired in NT but not TH rats. **(A)** NT rats spent less time with the familiar object than Sham rats. **(B)** NT but not TH rats moved faster, traveled farther, and spent less time immobile than Sham rats. **(C)** Female rats moved faster and traveled farther than male rats during the 2-min testing period. **(D)** Both NT and TH female rats moved with slower peak speeds than Sham female rats; TH female rats spent more time immobile than NT female rats. Horizontal lines represent mean **(A,B)** time immobile **(C,D)**, or median [**(B)** speed and distance]. Shaded bars show the standard deviation (mean graphs) or interquartile range (median). Dots represent individual rat values. *p < 0.05; **p < 0.01.

In this test, NT rats spent less time with the novel object than Sham rats (*t*-test: *p* = 0.0202). The time TH rats spent exploring the novel object fell between Sham and NT means but was not statistically different from either group (Figure 4A; *t*-test: *p* = 0.1838, 0.2499). This indicates recognition memory is impaired by HI-injury and somewhat improved by TH. During the 2-min testing time, NT but not TH rats moved faster, traveled farther, and spent less time immobile than Sham rats (Figure 4B; speed: KW H(2) = 6.8767, *p* < 0.0321; SD *p* = 0.0126, 0.6847; distance: KW H(2) = 7.1552 *p* < 0.0279; SD, *p* = 0.0099, 0.6847; time immobile: ANOVA F(2,26) = 4.1569, *p* = 0.0271; Dunnett’s test, *p* = 0.0320, 0.8281). These behaviors are characteristic of hyperactivity and difficulty in maintaining attention (Thompson et al., 2018). Since the TH mean fell between the Sham and NT means and only NT was significantly different from Sham, it appears that overall, TH somewhat attenuated hyperactive locomotion during movement in recognition memory testing. Stratifying these data by sex revealed female rats moved faster and traveled farther than male rats during the 2-min testing period (Figure 4C; *t*-test: *p* = 0.0084, 0.0090). Additionally, female NT and TH rats moved with slower peak speeds than female Sham rats (Figure 4D; ANOVA *F*(2,11) = 5.3136; Dunnett’s test: *p* = 0.0243, *p* = 0.0219, 0.0357), suggesting an impairment in locomotion speed during novel object exploration that was not improved by TH-treatment. However, TH-treated female rats spent more time immobile than female NT rats, indicating a possible improvement in attention by TH treatment (Thompson et al., 2018) (Figure 4D; ANOVA F(2,11) = 4.1451, *p* = 0.0455, Dunnett’s test: *p* = 0.0368).

### 3.3 Food protection

**Figure 5:**
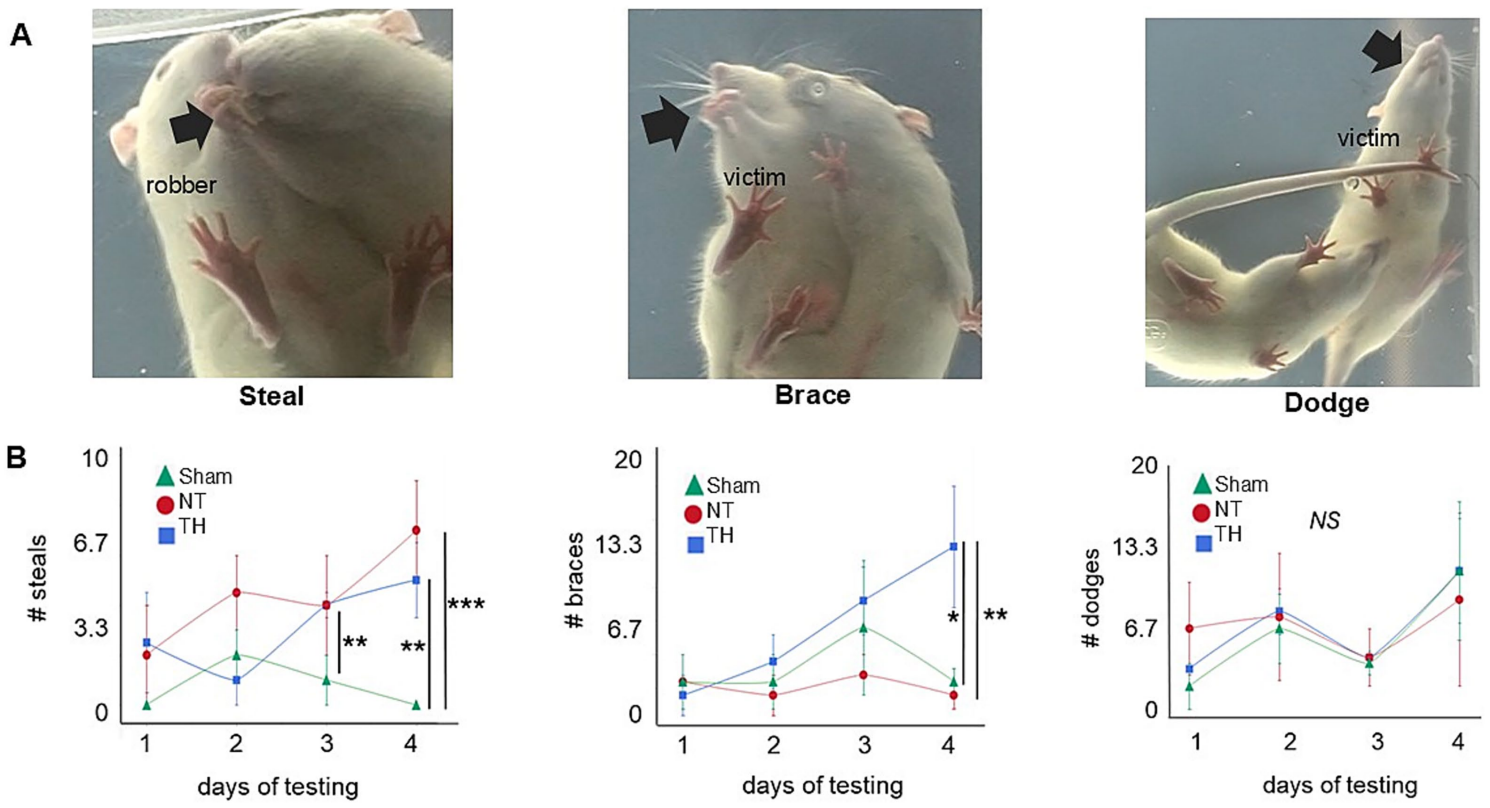
Food protection. **(A)** Video stills depict a representative (L–R) steal, brace, or dodge. The black arrow highlights the location of the food item: in a steal, the robber takes the item away from the victim rat; in a brace, the food item is braced with the victim’s forepaws as it turns away from the robber rat; and in dodge, the item is held in the victim rat’s mouth while all four paws are used to move away from the robber rat. **(B)** Number of each (L–R) steals, braces, and dodges over each of the four testing days (Greco et al., 2020; Thornton et al., 2017; Northington et al., 2011; Kebaya et al., 2023). Colored shapes indicate the median for each treatment group, and error bars represent the median absolute deviation. *p < 0.05; **p < 0.01; ***p < 0.001. The “**” symbol in day 3 steals refers to the significant difference between Sham and NT (*p* = 0.0100); the comparison between Sham and TH was not significant (*p* = 0.0569).

Both NT and TH rats had more food items stolen than Sham rats, over the four testing days (sum of days 1–4: KW H(2) = 16.5513, *p* < 0.0003; SD, *p* = 0.0035, 0.0175) and on day 4 of testing (Figures 5A,B; KW H(2) = 15.5720, *p* < 0.0004; Wilcox, *p* = 0.0009, 0.0022); this indicates the ability to successfully protect food from theft was impaired by HI-injury. Where the number of steals declined over the four testing days in Sham rats, they increased in NT and TH rats, suggesting HIE rats did not improve in their ability to protect food as rapidly as Sham animals.

Food protection behavior typically begins with dodges, then, as the size of the food item decreases, transitions to braces (Peters, 2018). The timing of this transition depends on the size of the food item. When a food item is first given to the victim rat, it is larger, and since the time needed to eat it is relatively long, the victim rat therefore engages in dodges to put a larger distance between itself and the robber rat (Whishaw and Gorny, 1994). As the food item decreases in size, the time needed to eat the item decreases, and the victim rat transitions to braces, since a considerable distance between itself and the robber is not needed (Peters, 2018). Here, the number of dodges appears somewhat uniform across treatments and over the four testing days. Braces, however, were consistently higher in TH rats and increased over the testing days compared to NT rats (Figure 5B; slope of braces days 1–4, Welch’s, *p* = 0.0037; GH, *p* = 0.0029). It is unclear if this increase in braces that are unique to TH-treated rats is a side effect of TH-treatment or a result of their fewer steals in conjunction with longer time spent engaging in the trials. TH rats engaged in day 4 trials 25.0% longer than NT rats, though this was not significant (Wilcox, *p* = 0.0991).

**Figure 6:**
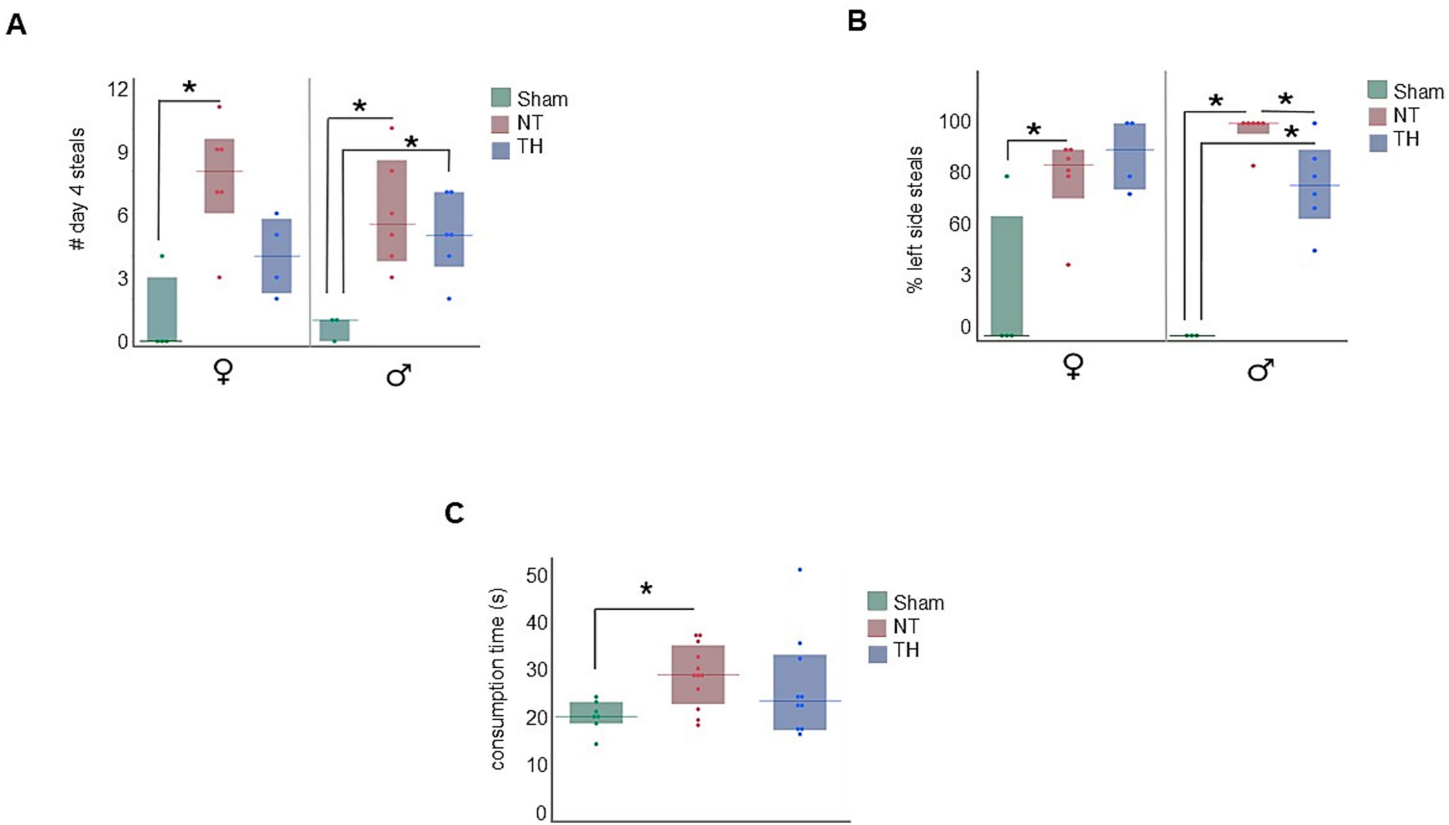
Additional food protection results. **(A)** Steals by treatment and sex. NT rats of both sexes demonstrated more steals than Sham rats on day 4, and TH female rats demonstrated a modest improvement in steals compared to NT female rats. **(B)** Depicts the percentage of D4 steals that occurred on the victim rat’s left or right by treatment and sex. Both male and female NT rats had more food items stolen from the left side of their body than Sham rats. The TH male but not female TH rats had less items stolen from the left side of their body than NT rats of their same sex. **(C)** NT but not TH rats took longer to chew their food items than Sham rats. *p < 0.05 > 0.01. Floating bars represent median values for each treatment, and solid bars represent the interquartile range. Dots represent individual rat values.

While NT rats of both sexes had more food items stolen from them than Sham rats, the results in TH-treated rats were sexually dimorphic. Male TH-treated rats had numbers of stolen food items similar to those of male NT rats, but steals from TH-treated female rats were not statistically different from Sham [(Figure 6A) male steals: KW H(2) = 7.0422, *p* < 0.0296; Wilcox, *p* = 0.0275 NT, 0.0262 TH; female steals: KW H(2) = 8.4357, *p* < 0.0147; Wilcox, *p* = 0.0226 NT, 0.1038 TH]. This suggests TH-treatment benefited females with HIE more so than males.

Since the right cerebral hemisphere in this cohort of rats is ipsilateral to ischemic injury, and motor deficit was observed in the contralateral forelimb in this cohort [previously published in Saadat et al. (2023) and demonstrated in Figures 3A,B], we stratified the number of steals to specify which side of the body the item was stolen from. In both male and female NT rats, more food items were stolen from the left side of their body [(Figure 6B) male KW H(2) = 10.5202, *p* < 0.0052; female KW H(2) = 6.0277, *p* < 0.0410]. However, a sexually dimorphic response was seen with TH-treatment, where the percent of left-side (ischemia side) steals in males, but not female rats, was somewhat improved by TH (Figure 6B; *p* = 0.0256, 0.0723). While no sex-specific difference in the type of food protection behavior used (e.g., braces and dodges) has been described in past publications, differences in how these behaviors are physically carried out have: females use their snout more and pivot with their lower body to protect items, where males pivot at middle of their body and take more steps to protect items (Peters, 2018). Meaning it could be that the improvement seen here in TH-treated males is due to motor, not cognitive improvement. When the robber rat was removed from the environment, NT but not TH rats took longer to consume a food item than Sham rats [median 28.6 s in NT rats compared to 20.3 s in Sham and 23.4 s in TH; Figure 6C; KW H(2) = 6.1738, *p* < 0.0456; Wilcox, *p* = 0.0160, 0.2225]. This demonstrates a probable feeding impairment from HIE, which is consistent with the feeding difficulties commonly seen in human children with HIE (Arora et al., 2022), and TH treatment modestly improved this deficit.

## 4 Discussion

Speed, distance, and immobility were all increased in injured rats during both the open field and NOR tests; together, these behaviors are indicative of hyperactivity (Thompson et al., 2018). Since hyperactivity is associated with disordered attention (Mueller et al., 2017), this seems to align with the difficulties in attention and executive functioning that are known sequelae of HIE seen in human outcomes (Halpin et al., 2022). Hippocampal lesions were observed in this cohort of rats as a consequence of HI-injury [previously published in Saadat et al. (2023) and shown here in Figure 3] and have been associated with increased locomotion and hyperactivity in rodents (Thompson et al., 2018); therefore, it is probable that injury to the hippocampus contributed to the hyperactive behaviors observed in this study.

Another behavior we observed in the HIE animals was an increase in turns toward the left side of the body (Figure 2C). Since the left side of the body is contralateral to the cerebral injury, this tendency could be a consequence of contralateral weakness and neglect. Weakness in the contralateral forelimb was observed in this cohort (Saadat et al., 2023) and to compensate for this weakness, the rats may be turning toward the contralateral side as an effort to obtain more sensory input, as well as to maintain balance and orientation. The cerebral asymmetry caused by ipsilateral tissue loss observed could, in turn, create vestibular dysfunction, which can also contribute to circuitous movement in rodents (Loscher, 2010).

In the food protection assay, rats with HIE were less capable of protecting food items than Sham rats, and modest improvement was seen with TH treatment (Figure 5B). This deficit in the ability to protect food items could be a consequence of cortical and hippocampal injury. Cerebral damage in this cohort was defined by unilateral tissue loss and injury to cortical, hippocampal, and thalamic regions, concentrated approximately −3 to −3.5 mm from bregma [previously published in Saadat et al. (2023) and shown here in Figure 3]. This cortical damage included both parietal and somatosensory areas [Figure 3A], and since unilateral damage to the parietal cortex was shown *not* to affect food protection behaviors in another study (Whishaw, 1988), somatosensory damage is the more likely contributor to this impairment. The functional contribution of the hippocampal injury to the increase in steals may be due to an inability to sustain or switch attention from the item to the robber (Martin et al., 2008). This could translate into challenges with multitasking, adapting to changing social situations, and maintaining focus on social interactions or tasks (Cainelli et al., 2021). Further supporting the ties to cerebral injury are the asymmetry in the side of the body from which the food items were stolen (Figure 6B). This observation is consistent with Whishaw and Tomie’s finding that unilateral cortical removal only impaired protecting items from the contralateral side (Whishaw and Tomie, 1988). Moreover, the number of steals worsened over time in injured rats (Figure 5B). This indicates learning deficits from HI-injury, and learning difficulties are a known consequence of human HIE (Schreglmann et al., 2020) and have been recapitulated in rodents (Maxwell et al., 2020). Survivors of HIE often struggle with processing and applying new information, affecting learning from experiences and adapting to social situations (Kromm et al., 2024).

Also in the food protection test, bracing behavior increased over the four testing days only in TH-treated rats, not in Sham or untreated HIE rats (Figure 5B). Typically, rats primarily use dodging behaviors to evade robbers, then transition to braces as the food item gets smaller. The transition from dodging to bracing behavior is thought to be driven by an internal clock: when the food item is first taken, the perceived time to eat the food item is longer, and the victim rat engages in more dodges (Whishaw and Gorny, 1994). As the item is consumed and becomes smaller, the perceived time to eat the food item decreases, and the victim braces more often (Peters, 2018). This unique increase in braces in TH rats suggests their internal clock is altered, causing them to transition from dodges to braces too early, and anterograde interference may explain this disruption. Taken together, the atypical food protection behaviors suggest impairments in anticipatory responses and temporal processing, as they resulted in the inability to successfully avoid food items being stolen. These deficits could manifest in HIE survivors as difficulties in social timing, turn-taking, and appropriate responses in social interactions, potentially leading to social awkwardness or isolation in adulthood (Cainelli et al., 2021).

## 5 Conclusion

Neonatal rats with mild–moderate HIE demonstrated impairments in attention, learning, and skill development, and processing egocentric cues. Treatment with the standard of care, TH, demonstrated sexual dimorphisms in protecting food items as well as speed and locomotion during exploration. These findings underscore the need for further characterization and understanding of the long-term social and cognitive impairments resulting from neonatal HIE, even in cases treated with therapeutic hypothermia. The cognitive deficits in attention, task-switching, and social interactions seen in this study may have profound implications for interpersonal relationships and adaptive functioning in survivors of neonatal HIE. Better understanding and characterizing the long-term cognitive impacts of HIE and other diseases of brain injury in animal studies can aid in the development of targeted interventions and help survivors better adapt to the social and cognitive demands of adulthood.

## Data Availability

The raw data supporting the conclusions of this article will be made available by the authors, without undue reservation.

## Glossary

Hypoxic–ischemic encephalopathy (HIE): Neonatal brain disease caused by temporary deprivation of oxygen and glucose at or around birth.
Therapeutic hypothermia (TH): Cooling therapy, standard of care treatment for HIE; also used in other diseases of hypoxic–ischemic injury (e.g., heart attack and stroke) and other clinical applications (e.g., to prevent hair loss after radiation treatment).
Novel object recognition test: Behavioral test where recognition memory is measured by the time spent exploring a novel object (NO) relative to a familiar object (FO) the animal has been exposed to previously.
Open field test: Behavioral test where locomotion and exploratory behaviors are observed and compared to control animals representing positive and negative injury; room lighting is usually dimmed to encourage exploration.
Food protection test: Behavioral test; a victim rat is given a food item, and behaviors used to protect the item are measured as it instinctively evades, or tries to evade, theft of the item by a robber rat.
Victim rat: In the food protection assay, the rat given the food item, which therefore tries to protect the item from theft by the robber rat.
Robber rat: In the food protection assay, the rat that is not given the food item and who therefore tries to steal it from the victim rat.
Dodge: Food protection behavior where the food item was held in the rat’s mouth while it used its limbs to travel away from the robber rat.
Brace: Food protection behavior where the food item was braced in the victim rat’s forepaws as it pivoted its body away from the robber rat.
Steal: Event in the food protection assay where the robber rat successfully takes the food item away from the victim rat.
Interval timing: Short time intervals (seconds), pertaining here to the timing between food protection behavior intervals of dodging to bracing behaviors.
Topographical disorientation: Impairment or inability to orient to one’s surroundings.
Anterograde interference: Learning difficulty where prior knowledge impedes learning new information.
