# Supplementary material for: Traditional and non-traditional behavioral tests demonstrate the attenuation of cognitive deficits by therapeutic hypothermia in a rat model of neonatal hypoxic–ischemic encephalopathy
Source: Front Behav Neurosci. 2025 Dec 17;19:1695435. doi: 10.3389/fnbeh.2025.1695435 (PMC12753503; doi:10.3389/fnbeh.2025.1695435)
Supplement: Supplementary file 1 [file Table_1.DOCX]

**Supplementary Table 1.** Statistical summary. Listed are the statistical tests used for each group and pair-wise comparison and their p-value results. For brevity, one-way analysis of variance was shortened to ANOVA, Kruskal-Wallis was abbreviated to KW, the Games-Howell post-hoc test was abbreviated to GH, and Steel-Dwass to S-D. “All” indicates statistical comparisons where sexes were pooled, and ♀ and ♂ symbols indicate sex-stratified comparisons. NA indicates a group comparison p-value >0.05.

| **Behavioral test** |  | **Group comparisons** | | **Treatment comparisons** | | | |
| --- | --- | --- | --- | --- | --- | --- | --- |
|  |  |  |  | **p-values** | | |  |
|  |  | **p-value or equivalent** | **Test used** | **Sham vs NT** | **Sham vs TH** | **NT vs TH** | **Test used** |
| ***Open field exploration*** | | | | | | | |
| Distance | All | 0.0287 | ANOVA | 0.0144 | 0.0138 | 0.9566 | GH |
| Mean speed | All | 0.0285 | ANOVA | 0.0140 | 0.0141 | 0.1719 | GH |
| Time mobile | All | 0.0184 | ANOVA | 0.0584 | 0.0179 | 0.8246 | GH |
| Anti-clockwise rotations | All | 0.0164 | KW | 0.0204 | 0.6792 | 0.1917 | S-D |
|  | **♀** | 0.0935 | KW |  | *NA* |  |  |
|  | **♂** | 0.1890 | ANOVA |  | *NA* |  |  |
| Clockwise rotations | All | 0.6331 | Welch’s |  | *NA* |  |  |
| # Progressions 0-300 s | All | 0.0387 | ANOVA | 0.1361 | 0.0265 | 0.6634 | GH |
| # Progressions 301-600 s | All | 0.3308 | ANOVA |  | *NA* |  |  |
| # Stops 0-300 s | All | 0.0255 | ANOVA | 0.0968 | 0.0220 | 0.6724 | GH |
| # Stops 301-600 s | All | 0.4421 | ANOVA |  | *NA* |  |  |
| ***NOR*** | | | | | | | |
| Time spent with FO | All | <0.2679 | ANOVA | *NA* | | | |
| Time spent with NO | All | 0.0632 | ANOVA | 0.0202 | 0.1838 | 0.2499 | T-test |
| Speed | All | 0.0321 | KW | 0.0126 | 0.6847 | 0.4797 | S-D |
| Distance | All | 0.0279 | KW | 0.0099 | 0.6847 | 0.4809 | S-D |
| Time immobile | All | 0.0271 | ANOVA | 0.0320 | 0.8281 | 0.0595 | Dunnett’s |
| Peak speed | **♀** | 0.0243 | ANOVA | 0.0219 | 0.0357 | 0.9998 | Dunnett’s |
| Peak speed | **♂** | 0.1779 | ANOVA |  | *NA* |  |  |
| Time immobile | **♀** | 0.0455 | ANOVA | 0.0595 | 0.7025 | 0.0368 | Dunnett’s |
| Time immobile | **♂** | 0.1259 | ANOVA | *NA* | | | |
| Mean speed ♀ vs. ♂ | | T-test | | ♀ vs. ♂ 0.0084 | | | |
| Distance ♀ vs. ♂ | | T-test | | ♀ vs. ♂ 0.0090 | | | |
| ***Food Protection*** | | | | | | | |
| Total behaviors day 1-4 | All | 0.0969 | ANOVA |  | *NA* |  |  |
| **Steals**  sum day1-4  day 1  2  3  4  4  4 |  |  |  |  |  |  |  |
|  | All | 0.0003 | KW | 0.0035 | 0.0175 | 0.0237 | S-D |
|  | All | 0.3211 | KW |  | *NA* |  |  |
|  | All | 0.0507 | KW |  | *NA* |  |  |
|  | All | 0.0156 | KW | 0.0010 | 0.0569 | 0.3346 | Wilcox |
|  | All | 0.0004 | KW | 0.0009 | 0.0022 | 0.0539 | Wilcox |
|  | **♀** | 0.0147 | KW | 0.0226 | 0.1038 | 0.0528 | Wilcox |
|  | **♂** | 0.0296 | KW | 0.0275 | 0.0262 | 0.6273 | Wilcox |
| Slope of steals day 1-4 | All | 0.0213 | ANOVA | 0.0250 | 0.1252 | 0.5922 | GH |
| %Left side steals day 4 | **♀** | 0.0410 | KW | 0.0299 | 0.0723 | 0.6670 | Wilcox |
|  | **♂** | 0.0052 | KW | 0.0141 | 0.0256 | 0.0263 | Wilcox |
| **Braces**  sum day 1-4  day1  2  3  4  Slope of braces 1-4 |  |  |  |  |  |  |  |
|  | All | 0.0158 | Welch’s |  | *NA* |  |  |
|  | All | 0.9912 | KW |  | *NA* |  |  |
|  | All | 0.0650 | KW |  | *NA* |  |  |
|  | All | 0.0676 | KW |  | *NA* |  |  |
|  | All | 0.0024 | KW | 0.0014 | 0.0274 | 0.1975 | Wilcox |
|  | All | 0.0037 | Welch’s | 0.5368 | 0.0028 | 0.0029 | GH |
| **Dodges**  sum day 1-4  day 1  2  3  4 |  |  |  |  |  |  |  |
|  | All | 0.7437 | ANOVA |  | *NA* |  |  |
|  | All | 0.0873 | KW |  | *NA* |  |  |
|  | All | 0.6389 | KW |  | *NA* |  |  |
|  | All | 0.7789 | KW |  | *NA* |  |  |
|  | All | 0.9682 | KW |  | *NA* |  |  |
| Slope of dodges 1-4 | All | 0.5739 | ANOVA |  | *NA* |  |  |
| Day 4 Consumption time | All | 0.0456 | KW | 0.0160 | 0.2225 | 0.2225 | Wilcox |
|  | **♀** | 0.2036 | ANOVA |  | *NA* |  |  |
|  | **♂** | 0.0908 | ANOVA |  | *NA* |  |  |
| ***Cerebral injury (coronal)*** | | | | | | | |
| % Hemispheric reduction |  | 5.9e-5 | Welch’s | 0.0003 | 0.0162 | 0.0079 | GH |
| % Cortical reduction |  | 0.0064 | Welch’s | 0.0074 | 0.1063 | 0.1259 | GH |
| % Hippocampal reduction |  | 5.2e-5 | KW | 0.0011 | 0.1825 | 0.0013 | S-D |
